# Making Contact

**Published:** 2014-04-28

**Authors:** Thomas R. Resch, Jonathan S. Lam, Stephen M. Milner, Leigh Ann Price

**Affiliations:** Johns Hopkins Burn Center, The Johns Hopkins University School of Medicine, Baltimore, Md

**Keywords:** electrical burn, high-voltage, hand, fasciotomy, burn

## DESCRIPTION

**Figure 1:**
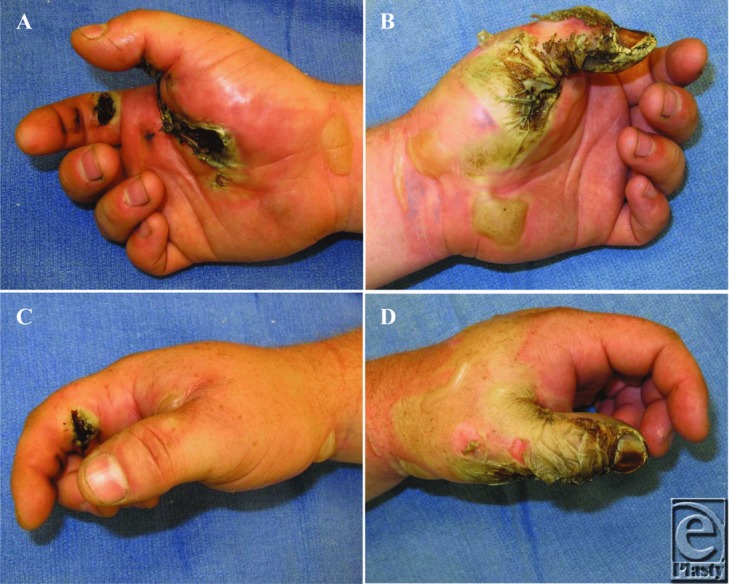
Two views of the right hand (A and C) and the left hand (B and D) following a high-voltage electrical injury.

A 31-year-old left-hand-dominant male was brought to the emergency department after contacting a high-voltage (7600 V) power line with a fiberglass extension pole. Physical examination revealed third-degree burns to the bilateral hands (Fig 1).

## QUESTIONS

1. **What is the definition of a “high-voltage” injury?**
2. **What are some common sources of electricity and what is the approximate voltage for each?**
3. **What other possible electrical injuries should the provider be aware of?**
4. **What is the surgical treatment?**

## DISCUSSION

High-voltage electrical injuries are often defined as being greater than 1000 V and low-voltage being less than 1000 V.^1^ This is a very important distinction because mortality and amputation rates typically increase 2- to 3-fold with high-voltage exposure.^2^ To clarify, the term “electrocution” refers only to death caused by electricity. In reality, the electrical current (amperage) rather than the voltage is the most important determinant of injury and death. Nevertheless, voltage was historically used to describe electrical injuries and continues to be used today.

The most common sources of electricity include household power outlets (120 V in the United States; 220 V in Europe and Asia), power lines (>100 000 V) and lightning (>10 million V).^1^ Interestingly, household static electricity can be well over 1000 V but does not have enough current to cause tissue damage.^3^^,^^4^

The electrical current in this case is predicted to have traveled from one hand to the other, crossing the torso. As a result, classic electrical injuries such as this may cause cardiac arrhythmias and deep tissue damage not evident by physical examination. To exclude cardiac injury, an admission electrocardiogram should be obtained, followed by continuous telemetry monitoring in select patients.^5^ Of note, both revealed normal findings in this case. Deep tissue damage can also cause rhabdomyolysis leading to pigment-induced acute kidney injury.^6^ Therefore, initial laboratory studies should include a creatine kinase level and urinalysis. Alternatively, a urine myoglobin level may be obtained, but it may be negative in up to half of patients with rhabdomyolysis.^7^ In this case, serial creatine kinase levels were followed closely while observing the trend and found to be only mildly elevated. Finally, less common but equally significant injuries to other organs such as nerves, blood vessels, bone, the gastrointestinal tract, and eyes can occur.^2^ Surveillance for long-term sequela such as cataracts and neuropathy must also be maintained.^2^

Surgical treatment may include decompressive fasciotomy to prevent compartment syndrome in addition to surgical exploration and serial debridement of nonviable tissue.^2^ In this case, the patient had neurologic complaints necessitating emergent bilateral hand fasciotomies and carpal tunnel release. Excisional debridement should be undertaken in stages, as deeper soft-tissue injuries may become evident over time. Application of splinting in position of function must be accomplished for preservation of joint use in the extremity and this should be commenced even prior to the first surgery.^2^ Despite serial debridement and attempted thumb salvage, amputation of the left thumb was ultimately required.

In summary, electrical injuries can be associated with extensive soft-tissue and multisystem organ damage that is not always evident by gross examination. Vigilance on the part of the provider is imperative for a successful patient outcome and long-term survival.
